# 低分化肺腺癌左上臂及背部肌内转移1例

**DOI:** 10.3779/j.issn.1009-3419.2012.04.11

**Published:** 2012-04-20

**Authors:** 江涛 陈, 征 田, 兴华 宋

**Affiliations:** 830054 新疆，新疆医科大学第一附属医院骨肿瘤科 Department of Bone Tumor, the First Affiliated Hospital to Xinjiang Medical University, Xinjiang 830054, China

肺癌晚期可出现各个不同脏器的转移而引起相应的症状，常常给患者带来极大的痛苦，甚至威胁到生命。肺癌转移主要以直接蔓延和淋巴结转移为主，晚期可发生血行转移。首发症状为软组织转移较少见，现对新疆医科大学第一附属医院诊治的1例低分化肺腺癌左上臂及背部肌内转移的病例报道如下。

## 1 病例资料

患者，男性，58岁，因发现左上臂及背部肿物1月余，于2011年12月5日入院治疗。患者1个月前无明显诱因出现左上臂和背部肿物各一个，既往无其它系统肿瘤病史。骨专科检查：左上臂触及一4 cm×3 cm×2 cm肿物，背部触及一4 cm×2 cm×2 cm肿物，均质韧，活动度差，与周围肌肉界限不清，形状欠规则，局部皮温正常，无静脉曲张。彩色超声多普勒检查(图 1)示左上臂局部肌层内可见大小约5 cm×2.5 cm×3.1 cm低回声区，距体表约0.3 cm，边界尚清楚，形态不规整，内回声不均匀，内可见丰富条索状血流信号，提示局部肌层内实性占位灶；背部局部皮下软组织内可见大小约4.4 cm×1.3 cm×2.2 cm低回声，距体表约0.7 cm，边界清晰，呈椭圆形，内回声尚均匀，可见少许点状血流信号。左上臂MRI(平扫+增强，图 2)检查提示左上臂肌内占位，恶性肿瘤可能。C12肿瘤芯片阳性结果：糖类抗原19-9为266.03 KU/L，糖类抗原242为 > 200 KU/L，铁蛋白为419.55 ng/mL，糖类抗原125为41.71 KU/L，糖类抗原153为35.64 KU/L。患者于2011年12月8日行左上臂及背部肿瘤扩大切除手术，术中见肿瘤组织与周围肌肉界限不清，质韧，将肿瘤组织从周围正常肌肉组织内游离后完整切除，术中探查见周围一肿大淋巴结并同送病理检查；术中背部所见情况同左上臂。术后病理行免疫组化检测(图 3)提示，AE1/AE3(+)，CAM5.2(+)，Vimentin(+)，S-100(+)，CK7(+)，EMA(+)，病理诊断为转移性低分化肺腺癌，淋巴结见癌转移。术后临床诊断为左上臂及背部低分化肺腺癌软组织转移。随后进一步行肺部高分辨CT检查(图 4)，提示两肺间质改变；右肺下叶肺大泡；左肺上叶后段胸膜下小结节；纵隔及双肺门多发趋向钙化淋巴结；双侧胸膜增厚。为进一步治疗，于切口拆线后转肿瘤内科进行化疗，左上臂及背部行局部放射治疗。目前随访中。

**1:**
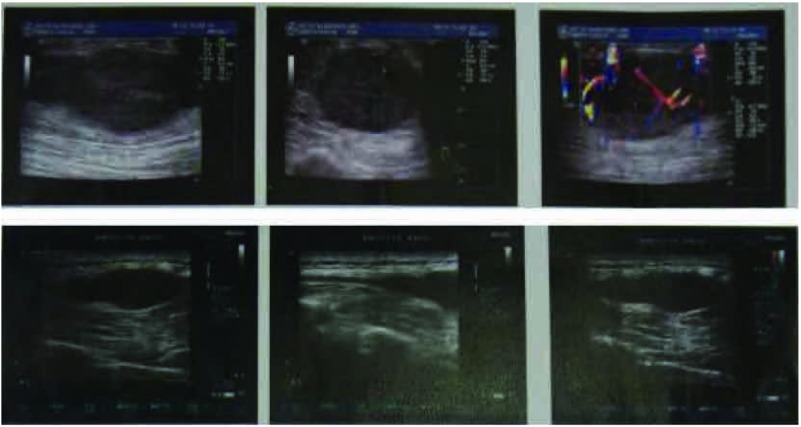
彩色超声多普勒检查示左上臂及背部软组织肿物 Color hepersound Doppler demonstrated masses in left arm and back soft tissue

**2:**
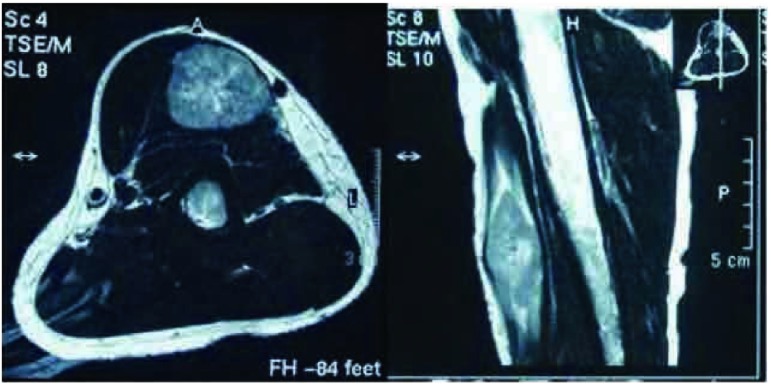
MRI检查示左上臂软组织肿物 MRI scan demonstrated a mass from soft tissue

**3:**
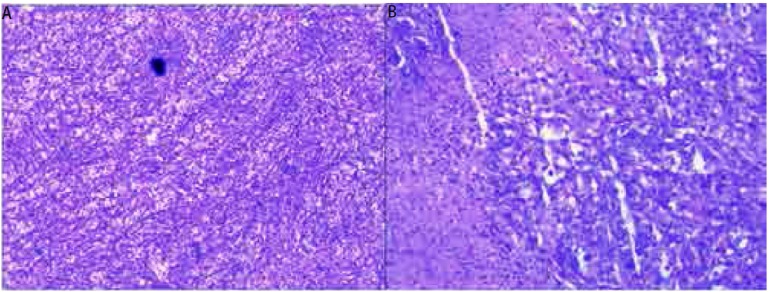
病理提示AE1/AE3(+)，CAM5.2(+)，Vimentin(+)，S-100(+)，CK7(+)，EMA(+)。A：免疫组化，×100；B：免疫组化，×400。 Pathological section demonstrated AE1/AE3(+), CAM5.2(+), Vimentin(+), S-100(+), CK7(+), EMA(+). A (Immunohistochemistry, ×100); B (Immunohistochemistry, ×400).

**4:**
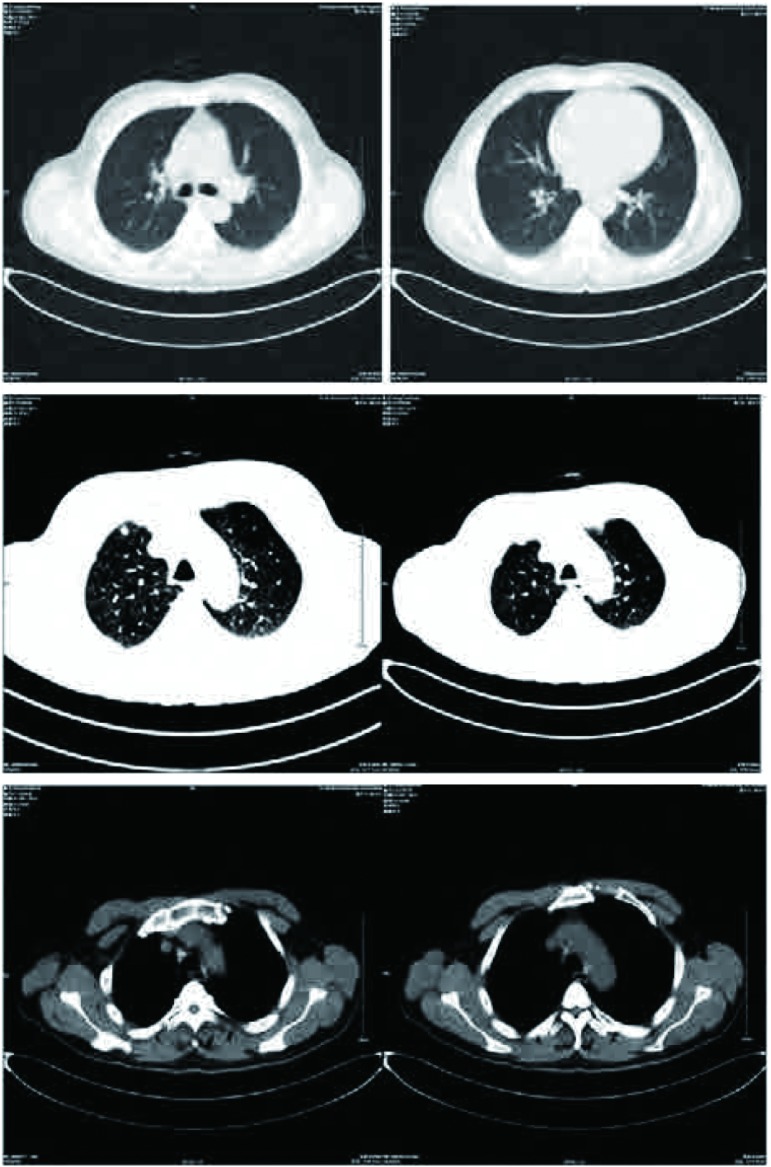
肺部高分辨CT示右肺下叶肺大泡，左肺上叶后段胸膜下小结节，纵隔及双肺门多发趋向钙化淋巴结，双侧胸膜增厚。 Chest CT scan demonstrated bullae in right lung lower lobe, a tubercle in posterior segment in left lung superior lobe, calcification lymph nodes in interpleural space and both hilus of lung and thickness in both membrana pleuralis.

## 2 讨论

恶性肿瘤晚期约有20%-70%出现骨转移，其中以肺癌、乳腺癌及前列腺癌最为常见，约占骨转移病例的80%。肺癌骨转移的好发部位以肋骨、椎骨常见^[1]^，其引起的骨与软组织疼痛或综合征多以肺癌的首发症状而出现，表现十分复杂，易误诊误治^[2, 3]^。PET/CT检查可发现肺癌所致的全身多处肌肉组织转移^[4]^。Mathis等^[5]^对174例肌肉组织的新生物行穿刺活检和免疫组化染色检测，结果显示穿刺活检和免疫组化检测对查找原发灶非常有帮助。本例患者通过完整切除肿瘤组织后行免疫组化染色检测，最终确诊原发灶来源于肺。

Banzo等^[6]^报道肺腺癌无症状肌肉转移目前的治疗主要以化疗和放疗为主，预后较差。于亮等^[7]^报道1例肺癌首发阴茎海绵体转移癌，给予阴茎部放疗及对症处理，3个月后患者死亡。本例患者软组织转移癌切除后尚未见其它部位的转移灶，目前仍然在跟踪随访中。本例患者在无任何其它部位转移的情况下发生两处软组织转移临床上罕见，因此软组织转移瘤和原发性软组织肉瘤应予以鉴别。肺癌软组织转移与其分化程度和病理类型密切相关，病理分化程度越低，肺外转移的发生率就越高。本例患者病理分级为低分化腺癌，老年男性，全身状况较好，术后给予化疗及局部辅助放射治疗，暂未发现其它部位转移。Ketata等^[8]^报道1例病例，55岁，男性，发现左上臂无痛性肿物，穿刺活检证实为肺癌，行化疗3个月后死亡。我们认为低分化的肺癌发生软组织转移，主要通过血行转移至远处的肌肉组织。鉴于软组织转移瘤与原发性软组织肉瘤治疗及预后截然不同，临床上出现的症状主要以受累部位的肿块出现为首发症状。因此临床医生采集临床病史时必须要详尽，软组织转移可能是肺癌患者的主要临床表现^[9]^；而病理医生首先要有整体观念和扎实全面的病理学知识，同时全面细致的检查能为鉴别诊断提供有价值的线索。
